# The complete mitochondrial genome of the *Gymnocypris chui* (Cypriniformes: Cyprinidae)

**DOI:** 10.1080/23802359.2017.1372702

**Published:** 2017-09-08

**Authors:** Jianshe Zhou, Chi Zhang, Wanliang Wang, Yingzi Pan, Benhe Zeng, Chaowei Zhou, Luo Lei, Baohai Li

**Affiliations:** a Fisheries Research Institute, Tibet Academy of Agricultural and Animal Husbandry Sciences, Lhasa, Tibet, People’s Republic of China;; b Department of Aquaculture, Southwest University Rongchang Campus, Chongqing, People’s Republic of China

**Keywords:** The *Gymnocypris chui*, mitochondrial genome, phylogenetic

## Abstract

The *Gymnocypris chui*, a new recorded species in Lange Lake, was grouped into genus *Gymnocypris* in Schizothoracinae, and had the rare quantity and limited resources on biology and genetics, especially in the mitochondrion. In this study, the complete mitochondrial sequence of *G. chui* was assembled and phylogenetic relationships with other species in Cyprinidae were analyzed. The whole mitochondrial sequence was 16,864 bp in length, which contained two control regions (D-loop regions), two rRNA genes (12S and 16S rRNA), 13 protein-coding genes and 22 tRNA genes. The D-loop region was separated by *tRNA^Pro^*. The 12S rRNA and 16S rRNA located between *tRNA^Phe^* and *tRNA^Leu^* and were separated by *tRNA^Val^*. The 13 mRNAs had three start codons, five termination codons and four overlap regions. The 22 tRNA scattered among the whole mitochondrion, and they were range from 66 (*tRNA^Cys^*) to 76 (*tRNA^Lys^* and*tRNA^Leu^*) in length. To further explore the phylogenetic relationship of the *G. chui*, we constructed the phylogenetic tree and verified that the *G. chui* was a part of genus *Gymnocypris* and had closer relationship with *Gymnocypris dobula* and was independent from other species of Schizothoracinae, Barbinae and Labeoninae in Cyprinidae. This study provided the valuable evidence on phylogenetic relationship of the *G. chui* at the molecular level and essential resource for further research on this species.

The unique geography and climate characteristics of Tibetan Plateau provided us to explore the phylogenetic relationships among species (He and Chen 2007), including fish. The *Gymnocypris chui*, a new recorded species in Lange Lak, was grouped in genus *Gymnocypris* in Schizothoracinae (Yang and Huang 2011). The chromosome number of *G. chui* was same with the *Gymnocypris. scleracanthus* (2n = 92) (Zhang et al. 2016). This freshwater fish mainly fed with zooplankton, hydrophyte and algae, and was treated as weakfish in Tibet. Nevertheless, the biology and genetics resources were limited to explore the molecular mechanism of *G. chui*, especially mitochondrion. Then the complete mitochondrial sequence of *G. chui* was assembled and phylogenetic relationships with other species in Cyprinidae were analyzed in this study.

The sample of *G. chui* was collected from Lange Lake (29°12′33.45″N 87°23′5.46″E). The genomic DNA was extracted by the DNeasy Tissue Kit (Qiagen, Germany), following the standard procedure. Total DNA was stored at −80 °C immediately until next step was conducted. The sequence was amplified by PCR with fifteen pairs of primer. In general, the length of this sequence is 16,771 bp (Genbank No: MF459673), containing two control regions (D-loop regions), two rRNA genes (12S and 16S rRNA), 13 protein-coding genes and 22 tRNA genes.

The D-loop regions located between *tRNA^Thr^* and *tRNA^Phe^*and were separated by *tRNA^Pro^*, consisted with the *Garra. kempi* (a species of genus *Garra*in Labeoninae in Cyprinidae) (Li et al. 2016). The whole D-loop region was 1112 bp in length. The 12S rRNA (960 bp) and 16S rRNA (1682 bp) located between *tRNA^Phe^* and *tRNA^Leu^* and were separated by *tRNA^Val^*. Among the 13 protein-coding genes, ATP8 (165 bp) took the shortest sequence and ND5 (1.824 bp) took the longest, consisted with previous studies (Noack et al. 1996; Peng et al. 2006; Li et al. 2016). Besides, 11 genes took thes tart codon of ATG, while *COX1*and *ND6*got GTG and TTA, respectively. The termination codon of these 13 protein-coding genes had five types, including CAT in *ND6*, AGG in *ND4*, “T- -” in*CYTB*, TAG in four genes (*ND1*, *ND2*, *ND3* and *ATP8*) and TAA in six genes (*ND4L*, *ND5*, *ATP6*, *COX1*, *COX2* and *COX3*). The 22 tRNA scattered among the whole mitochondrion, and they were range from 66 (*tRNA^Cys^*) to 76 (*tRNA^Lys^* and *tRNA^Leu^*) in length. Furthermore, four overlaps between protein-coding genes were found, including the *ATP6* and *ATP8* with 7 bp, *ATP6* and *COX3* with 1 bp, *ND4* and *ND4L* with 7 bp, *ND5* and *ND6* with 4 bp, which was similar with other species in Cyprinidae (Li et al. 2016; Zhou et al. 2016).

The *G. chui*, a species of genus *Gymnocypris* belongs to Schizothoracinaein Cyprinidae. To explore the phylogenetic position and relationship of the *G. chui*among other species among different subfamily in Cyprinidae, the complete mitochondrion sequences of 19 species of five genus from three subfamilies in Cyprinidae and the complete mitochondrion sequence of the *G. chui*in this study were used to construct the phylogenetic tree by MEGA6.06 software with ML analysis, and *Huso huso* (Acipenseridae, Acipenseriformes) and *Plotosus lineatus* (Plotosidae, Siluriformes) were taken as the outgroup (Figure 1). The neighbour-joining (NJ) tree (with 1000 bootstrap replicates) and the bootstrap of each cluster verified that the *G. chui*belonged to genus *Gymnocypris*and had closer relationship with *Gymnocypris dobula* than with other species of Gymnocypris (*G. namensis*, *G. przewalskii ganzihonensis* and *G. eckloni*). Meanwhile, the *G. chui* was independent from Schizothorax (*S. chongi*, *S. kozlovi*, *S. lantsangensis*, *S. labiatus*, *S. progastus* and *S. pseudoaksaiensis*), Acrossocheilus (*A. barbodon*, *A. paradoxus*, *A. fasciatus* and *A. wenchowensis*), Osteochilus (*O. hasseltii* and *O. salsburyi*) and Garra (*G. imberba*, *G. kempi* and *G. orientalis*). The Gymnocyprisand Schizothoraxwere a part of the Schizothoracinae, the Acrossocheilus was in the Barbinae, the Osteochilus and Garra belonged to the Labeoninae, which all classified to the Cyprinidae. This result provided the valuable evidence on phylogenetic relationship of the *G. chui* at the molecular level.

**Figure 1.**
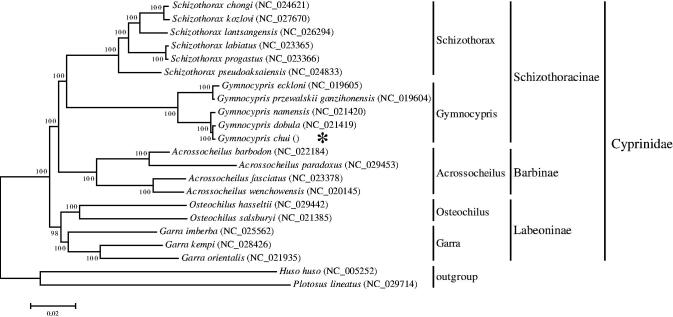
A neighbour-joining (NJ) tree of the *Gymnocypris scleracanthus* was constructed using mitogenome sequences. The phylogenic tree is constructed by Kimura 2-parameter method with 1000 bootstrap replicates. GenBank accession numbers of mitogenomic sequences for each taxon are shown in parentheses.
